# Repurposing Atorvastatin, HMGCO-A Reductase Inhibitor, in Patients with Ulcerative Colitis: A Randomized Controlled Study

**DOI:** 10.3390/jcm14093077

**Published:** 2025-04-29

**Authors:** Hayam Ali AlRasheed, Sahar M. El-Haggar, Sahar K. Hegazy, Maha M. Maher, Monir M. Bahgat, Mostafa M. Bahaa

**Affiliations:** 1 Department of Pharmacy Practice, College of Pharmacy, Princess Nourah bint Addulrahman University, Riyadh 84428, Saudi Arabia; 2 Clinical Pharmacy Department, Faculty of Pharmacy, Tanta University, El-Guiesh Street, El-Gharbia Government, Tanta 31527, Egypt; 3 Pharmacy Practice Department, Faculty of Pharmacy, Horus University, New Damietta 7952567, Egypt; 4 Internal Medicine Department, Faculty of Medicine, Horus University, New Damietta 7952567, Egypt; 5 Internal Medicine Department, Faculty of Medicine, Mansoura University, Mansoura 35516, Egypt

**Keywords:** atorvastatin, disease activity index, ulcerative colitis, myeloperoxidase, zonulin

## Abstract

**Background/Objective**: Among the inflammatory bowel illnesses, ulcerative colitis (UC) affects 5 million people worldwide. UC manifests as weight loss, rectal bleeding, persistent diarrhea, and abdominal pain. Experimental research focused into the potential benefits of atorvastatin for colitis, although the literature only has a small amount of clinical evidence. To examine atorvastatin’s protective effect in UC patients by assessing its impact on fecal myeloperoxidase, zonulin, and disease activity index (DAI). **Methods**: Two groups of patients with mild to moderate UC were randomly assigned. Over a six-month period, the control group (placebo group) received a placebo alongside mesalamine (1 g, three times daily [t.i.d.]). The atorvastatin group received atorvastatin (80 mg once daily) in addition to mesalamine (1 g t.i.d.). Disease severity was assessed by a gastroenterologist using the Disease Activity Index (DAI). Serum zonulin and fecal myeloperoxidase levels were measured before and after treatment to assess the biological efficacy of the interventions. Outcomes: Reduction in DAI and biomarker levels. **Results**: Both groups showed a significant decrease in DAI, zonulin, and fecal myeloperoxidase levels. However, the atorvastatin group (n = 23) demonstrated a significantly greater decrease in zonulin (*p* = 0.04), fecal myeloperoxidase (*p* = 0.03), and DAI (*p* = 0.001) compared to the placebo group (n = 24). In atorvastatin group, a significant correlation was observed between DAI and zonulin (*p* = 0.007, r = 0.4) and myeloperoxidase (*p* = 0.02, r = 0.36). **Conclusions**: The co-administration of atorvastatin may serve as a potential adjunct therapy for patients with UC.

## 1. Introduction

Chronic inflammatory diseases of the gastrointestinal tract that alternate between periods of remission and recurrence are referred to as inflammatory bowel disease (IBD), which includes Crohn’s disease (CD) and ulcerative colitis (UC). Diarrhea, rectal bleeding, and abdominal pain are common manifestations. From the mouth to the anus, inflammation in CD can impact each part of the digestive system, whereas in UC, inflammation is typically restricted to the colon (large intestine) and rectum [1]. Although the exact etiology remains unclear, research suggests that a combination of genetic, immunoregulatory, and environmental elements contribute to the pathogenesis of this abnormal autoimmune disorder [2]. CD is characterized by transmural ulceration that can affect any part of the gastrointestinal tract, with the terminal ileum and colon being the most commonly involved sites. Both CD and UC are classified based on disease severity (mild, moderate, or severe) and anatomical location [3]. CD is characterized by patchy inflammation, granuloma formation, fistula development, and deeply penetrating ulcers. In contrast, UC is limited to the colon and rectum, presenting with continuous mucosal inflammation and uniform lesions [4].

Ulcerative colitis is an idiopathic, chronic inflammatory illness that causes widespread inflammation of the colon and rectal mucosa. However, little is known about the precise underlying mechanisms [5]. Additionally, novel therapies that delay the progression of colon cancer and enhance results are required due to its high risk [6]. The pathophysiology of UC is influenced by multiple factors, including immune dysregulation, genetic predisposition, epithelial barrier dysfunction, and environmental triggers. Increased gut permeability and elevated inflammatory mediators in the inflamed mucosa are strongly associated with IBD [7,8].

In response to infection, the body activates its defense mechanisms by initiating inflammation, a process characterized by increased immune activity in the affected areas. This response can manifest through symptoms such as swelling, pain, or bruising [9]. In healthy individuals, the immune system resolves inflammation once a threat is neutralized, restoring the body to a normal, uninflamed state. However, in IBD patients, gut inflammation persists once triggered, requiring external intervention for resolution. The condition is particularly challenging due to its unpredictable flares and the discordance between symptoms and underlying inflammation [10].

The focus of management has changed to a treat-to-target strategy that requires knowledge of inflammatory activity in order to accomplish both clinical remission and mucosal repair. The techniques currently employed to assess inflammation in IBD have significant limitations. Laboratory tests and imaging/endoscopy are now the two primary methods used to diagnose and monitor IBD [11]. Cytokines, essential chemical messengers in cell signaling, play a crucial role in regulating inflammation. In IBD, an imbalance between pro- and anti-inflammatory cytokines influences disease onset, recurrence, and severity. Markers of immune and inflammatory activity serve as promising targets for non-invasive disease monitoring. Blood and stool are the primary biofluids used for tracking these indicators [12]. Because they shed in the intestinal lumen, activated leukocytes can be seen in feces after infiltrating the mucosa. However, because neutrophils have a short lifespan, determining their presence in the feces is insufficient; thus, the sample should be evaluated within a few hours of collection. After moving to the gut mucosa, neutrophils in an acute exacerbation release chemicals like myeloperoxidase into the gut lumen [13]. Therefore, fecal myeloperoxidase estimation serves as a potential biomarker for assessing intestinal inflammation in patients with UC [1]. Zonulin is a key regulator of intestinal permeability, modulating tight junction integrity between enterocytes [14]. In patients with UC, elevated serum zonulin levels have been associated with impaired intestinal barrier function, contributing to increased mucosal inflammation and disease severity [15]. Dysregulated zonulin expression may allow luminal antigens and pathogens to translocate across the gut epithelium, triggering immune responses that exacerbate colonic inflammation. As such, zonulin is increasingly recognized as a potential biomarker for intestinal barrier dysfunction and disease activity in UC [16].

A class of antihyperlipidemic medications known as 3-hydroxy-3-methylglutaryl coenzyme A (HMG-CoA) reductase inhibitors, or statins, are frequently used to treat hypercholesterolemia in individuals with atherosclerotic and coronary artery disorders [17]. Accumulating evidence suggests that statins exert beneficial effects on cardiovascular diseases, primarily due to their pleiotropic anti-inflammatory and immunomodulatory characteristics [18]. Two studies have shown that pravastatin and rosuvastatin mitigate intestinal inflammation severity in mouse models of dextran sulfate sodium (DSS)-induced colitis [19,20]. Furthermore, research has shown that atorvastatin has immunomodulatory effects in colitis-like mice models [21,22,23,24]. It is interesting to note that prior research has shown that simvastatin reduces colitis via blocking nuclear factor Kappa B (NF-κB) activation [25]. In oxazolone-induced colitis, El-Mahdy et al. found that atorvastatin and mesalamine markedly decreased zonulin levels, decreased the disease activity index (DAI), and increased tight junction proteins [26]. In mild to moderate patients with UC, mesalamine in combination with metformin, fenofibrate, and pentoxifylline also considerably decreased inflammation and alleviated symptoms [27,28,29]. Until now, there are limited data about the use of atorvastatin in patients with UC.

Based on these findings, the present study aimed to determine whether the combination of mesalamine and atorvastatin could slow the progression of UC and investigate the potential mechanisms behind these beneficial effects.

## 2. Patients and Methods

This study was a continuation of our previously published work about the effect of atorvastatin in UC [30]. A total of 56 participants who satisfied the inclusion criteria were included in the trial between January 2023 and March 2024. The Mansoura University Faculty of Medicine’s Institutional Review Board provided ethical permission (authorization code: MDP.22.08.107). The Helsinki Declaration and its later amendments’ guiding principles were followed throughout the study’s execution. Every participant provided written informed consent. Participants received complete information regarding their freedom to leave the study at any time without facing any sanctions.

### 2.1. Inclusion Criteria

Both male and female patients were included in the study. Female participants were required to use effective contraception and provide a negative pregnancy test before enrollment. The study specifically included patients previously diagnosed with UC through colonoscopy and undergoing mesalamine therapy.

### 2.2. Exclusion Criteria

Patients with severed-type UC, immunosuppressive drugs, or systemic or rectal steroids were not included. Additionally, in order to avoid the metabolic effects of atorvastatin, patients with liver or kidney disease were not included. Additionally excluded were those with a history of total or partial colectomy, colon cancer, musculoskeletal disorders, or hyperlipidemia. Finally, patients having a history of medication allergies were not included in the study.

### 2.3. Study Design

**Figure 1:**
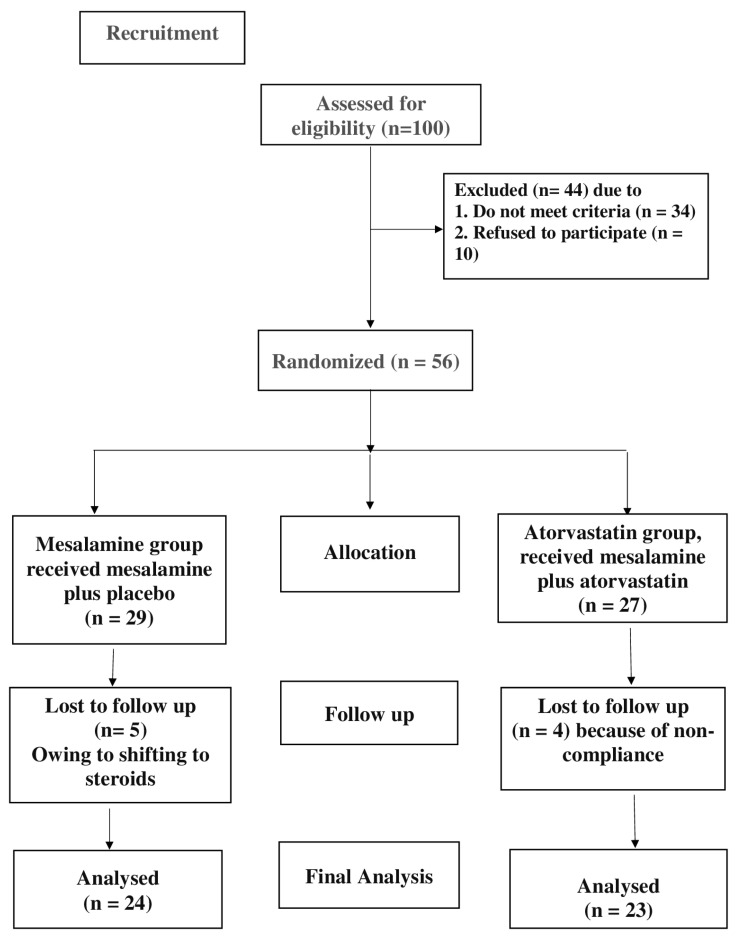
CONSORT diagram showing the flow of patients during study.

In 2022, the research study was formally registered on ClinicalTrials.gov with the registration number NCT05561062. As highlighted in the CONSORT flow diagram (Figure 1), participants were randomly assigned to one of two study groups. The allocation process was conducted using a computer-generated permuted block randomization method. Medication was dispensed by an unblinded pharmacist who had no role in outcome assessment. Both patients and physicians remained unaware of the treatment assignments to ensure blinding. The purpose of this double-blind, randomized controlled study was to assess the therapeutic effectiveness and safety of combining mesalamine and atorvastatin together to treat UC.

Written informed agreement was obtained from eligible patients, who were then randomized to one of two study groups. 

Group 1 served as the control group, receiving 1 g mesalamine tablets t.i.d. along with a placebo for six months (Pentasa^®^ 500 mg, Multi Pharm, Cairo, Egypt). 

Group 2 received 1 g mesalamine tablets t.i.d. in combination with 80 mg atorvastatin tablets once daily for six months (Ator^®^ 80 mg, EPICO, Cairo, Egypt).

### 2.4. Sample Size Calculations

No prior study has established the precise effect size of atorvastatin on changes in the DAI. Therefore, this research was designed as a pilot study, following the recommendations of Sima and Lewis, ref. [31] who suggested a minimum of 22 participants per group to detect a small to medium effect size while minimizing overall sample size. A total of 56 patients were randomized between the two groups, assuming a two-tailed α-error of 0.05, a statistical power of 80%, and accounting for a 20% dropout rate.

### 2.5. Study Protocol

Beyond eligibility screening, participants underwent comprehensive psychological, mental, and physical evaluations. They were then randomly assigned to one of two treatment groups: the placebo group, receiving 1 g of mesalamine tablets t.i.d. alongside a placebo, or the atorvastatin group, receiving 1 g of mesalamine t.i.d. in combination with 80 mg of atorvastatin once daily. Zeta Pharma Company produced placebo tablets that looked exactly like atorvastatin tablets in order to preserve blindness. All medications were administered orally, accompanied by nutritional and lifestyle counseling. References of mesalamine and atorvastatin were [32,33], respectively, based on previous studies.

### 2.6. Follow-Up

Follow-up was conducted through monthly phone calls and in-person meetings to monitor compliance and assess any reported side effects. During the initial visit, a comprehensive medical history was obtained, and laboratory tests, including liver and kidney function assessments and complete blood counts, were performed to exclude any underlying organic abnormalities. Additionally, serum zonulin and fecal myeloperoxidase levels were measured as biomarkers for intestinal inflammation.

### 2.7. Evaluation of Colitis

Following the methodology of Mitsuru Seo et al. (1992), the DAI was calculated for each patient both at baseline and after six months of treatment [34].

The DAI was calculated using the following formula: 13 × bowel movements + 60 × blood in stool + 0.5 × ESR − 4 × hemoglobin − 15 × albumin + 200. Index values below 150 corresponded to mild disease, values between 150 and 220 indicated moderate disease, and values above 220 reflected severe disease.

- Bowel movements: Indicates diarrhea frequency, serving as a measure of disease severity.
- Blood in stool: A key marker of mucosal inflammation and ulceration.
- Erythrocyte Sedimentation Rate (ESR): Reflects systemic inflammation levels.
- Hemoglobin: Assesses the impact of chronic inflammation and potential blood loss.
- Serum albumin: Evaluates nutritional status and disease severity.
- Constant factor (200): Applied for standardizing the scale.

### 2.8. Therapeutic Assessments

Therapeutic evaluation was performed by assessing DAI, serum zonulin, and fecal myeloperoxidase levels.

### 2.9. Sample Collection

Before the study commenced and again six months after treatment, 10 mL of blood was drawn from the antecubital vein. The collected blood was gradually transferred into test tubes, allowed to coagulate, and then centrifuged at 4500× *g* for 10 min using a Hettich Zentrifugen EBA 20 centrifuge, Westphalia, Germany. Two serum aliquots were prepared: one for routine kidney and liver function tests and the other stored at −80 °C for later cytokine analysis.

Fecal samples were weighed, dissolved in normal saline, and centrifuged. The clear supernatant obtained was used for myeloperoxidase analysis in fecal material.

### 2.10. Biochemical Analysis

Serum zonulin (catalog no.: 201-12-5578) and fecal myeloperoxidase (catalog no.: 201-12-0881) levels were quantified using commercially ELISA kits, following the manufacturer’s recommendations. The kits’ materials were provided by Sunredio, Shanghai, China.

The human myeloperoxidase concentration in samples was assessed using a double-antibody ELISA kit. Myeloperoxidase was first introduced into a monoclonal antibody-coated enzyme well, pre-treated with a human myeloperoxidase monoclonal antibody for incubation. Subsequently, biotin-labeled myeloperoxidase antibodies were added, which then bound to Streptavidin-HRP, forming an immune complex. After incubation and washing to remove unbound components, chromogen solutions A and B were introduced. The reaction caused a color shift from blue to yellow due to acid exposure, allowing for optical density measurement.

Serum zonulin was measured using a similar ELISA protocol.

### 2.11. Statistical Analysis

Statistical analysis was conducted using GraphPad Prism version 9 (GraphPad Software, Inc., San Diego, CA, USA). The Shapiro–Wilk test was applied to assess the normality of continuous variables.

For within-group comparisons, the Wilcoxon test was used for nonparametric data, while the paired Student’s *t*-test was applied to parametric data. Between-group comparisons were performed using the Mann–Whitney U test for nonparametric variables and the unpaired Student’s *t*-test for parametric variables.

Quantitative data were expressed as mean ± standard deviation (SD), medians, and interquartile ranges (IQRs) while qualitative variables were represented as numbers. The Spearman correlation test was employed to assess relationships between parameters. 

For categorical variables, the Chi-square test and Fisher’s exact test were applied. All *p*-values were two-tailed, and statistical significance was defined as *p* < 0.05.

## 3. Results

### 3.1. Clinical and Demographic Characteristics

The clinical, demographic, and laboratory data of placebo group or atorvastatin group were reported. These data included variables such as gender distribution (males/females), body mass index (BMI), liver function enzymes, alanine aminotransferase (ALT) and aspartate aminotransferase (AST), hemoglobin (Hgb), serum albumin, UC disease duration, number of smokers, and serum creatinine (Sr Cr). Lipid profile also was also reported between the two groups such as total cholesterol (TC), low density lipoprotein cholesterol (LDL), triglycerides (TG), and high-density lipoprotein (HDL). At the beginning of the study, no statistically significant differences were observed between the atorvastatin and placebo groups across all baseline variables (*p* > 0.05). The demographic characteristics and laboratory parameters, including BMI (*p* = 0.242), age (*p* = 0.759), sex (*p* = 0.599), AST (*p* = 0.194), hemoglobin (*p* = 0.209), ALT (*p* = 0.474), albumin (*p* = 0.479), SrCr (*p* = 0.186), TG (*p* = 0.391), LDL (*p* = 0.590), TC (*p* = 0.411), and HDL (*p* = 0.471), were comparable between both groups (Supplementary Materials Table S1).

Throughout the study, four patients from the atorvastatin group were lost to follow-up, while five from the placebo group were withdrawn. In the end, 47 patients completed the trial, and statistical analyses were performed per protocol on all measured parameters. The baseline demographic data, which are identical to those in our previously published study [30], have been included in the Supplementary Materials section (Supplementary Materials Table S1).

### 3.2. Effect of Study Drugs on Disease Activity Index

Table 1 presents a comprehensive analysis of clinical markers, including symptoms and disease activity scores, to evaluate the therapeutic impact of mesalamine alone versus mesalamine combined with atorvastatin in patients with UC. Baseline measurements for the DAI were statistically comparable between the two groups (*p* > 0.05), confirming similar disease severity at study initiation.

Treatment with mesalamine alone led to a statistically significant improvement in clinical parameters, with reductions in DAI (*p* < 0.0001), diarrhea score (*p* = 0.001), bleeding score (*p* = 0.003), and ESR (*p* < 0.0001), as well as significant increases in serum albumin (*p* = 0.03) and hemoglobin levels (*p* = 0.004), indicating the effectiveness of standard therapy.

Importantly, the group receiving the combination of mesalamine and atorvastatin showed significantly greater improvements from baseline across nearly all clinical outcomes. Compared to the mesalamine-only group, post-treatment values demonstrated more pronounced reductions in DAI and associated symptoms. Between-group analysis using the Mann–Whitney test revealed statistically significant differences in favor of the combination therapy for diarrhea score (*p* = 0.04), bleeding score (*p* = 0.03), ESR (*p* = 0.012), and hemoglobin (*p* = 0.003), while serum albumin did not show a significant between-group difference (*p* = 0.986). These findings underscore the enhanced therapeutic effect of the combination therapy in improving clinical parameters beyond the benefit observed with mesalamine alone.

### 3.3. Effect of Studied Drugs on Biological Markers

Table 2 presents the effects of the study medications on key serum inflammatory markers in patients with UC. At baseline, serum levels of zonulin and myeloperoxidase were comparable between the mesalamine-only and the mesalamine-plus-atorvastatin groups (*p* > 0.05), confirming similar inflammatory status before treatment. Both groups showed a statistically significant reduction in these markers following their respective treatments (*p* < 0.05), indicating within-group improvements. However, direct post-treatment comparisons using the Mann–Whitney test revealed that patients receiving the combination of atorvastatin and mesalamine had significantly lower levels of zonulin (*p* = 0.04) and myeloperoxidase (*p* = 0.03) compared to those treated with mesalamine alone. These findings reinforce the superior anti-inflammatory effect of the combination therapy over mesalamine monotherapy.

### 3.4. Correlation Analysis Between the Studied Parameters

**Figure 2:**
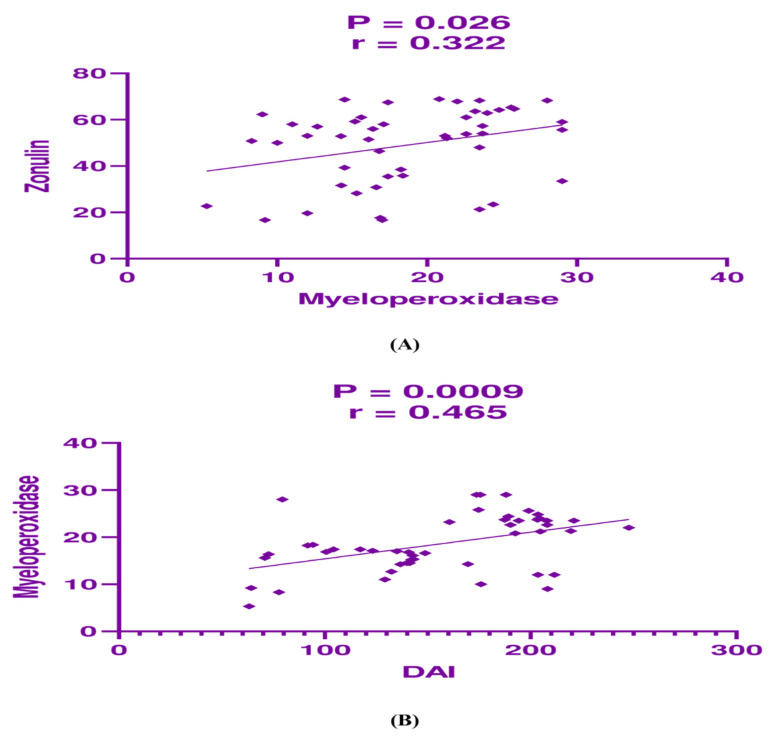
Correlation analysis between (**A**) zonulin and myeloperoxidase, and (**B**) myeloperoxidase and disease activity index (DAI) in mesalamine group.

In the mesalamine-only group, zonulin exhibited a significant but weak positive correlation with myeloperoxidase (*p* = 0.026, r = 0.322; Figure 2A) and a significant moderate positive correlation between myeloperoxidase and the DAI (*p* = 0.0009, r = 0.465; Figure 2B).

**Figure 3:**
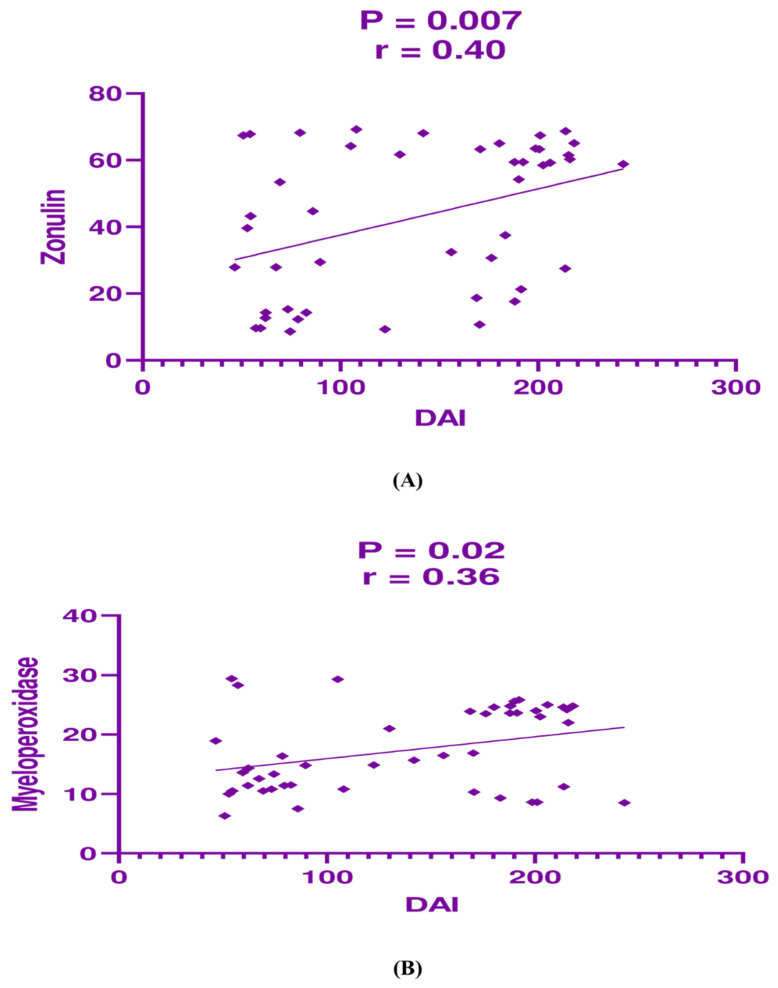
Correlation analysis between (**A**) disease activity index (DAI) and zonulin, and (**B**) DAI and myeloperoxidase in the combination treatment group.

In contrast, in the group receiving the combination of mesalamine and atorvastatin, DAI demonstrated a significant moderate positive correlation with zonulin (*p* = 0.007, r = 0.400; Figure 3A) and a significant weak positive correlation with myeloperoxidase (*p* = 0.020, r = 0.360; Figure 3B). These findings suggest that the relationship between inflammatory and barrier function markers may differ between monotherapy and combination therapy, underscoring the potential added benefit of atorvastatin.

## 4. Discussion

The large intestine is affected by the chronic inflammatory disease known as ulcerative colitis (UC). Despite significant advancements in treatment, including the introduction of immunomodulators and biological agents, managing UC remains challenging. Currently, no definitive cure exists for the disease, and available therapies primarily aim to suppress and regulate the inflammatory response to alleviate symptoms and prevent disease progression [35]. A range of anti-inflammatory and immunosuppressive agents are employed to manage CD and UC. However, these treatments often pose challenges, including immunosuppression, increased susceptibility to secondary infections, and cases of non-responsiveness, leading to persistent inflammation and worsening disease severity. As a result, the search for more effective and targeted therapies has been a key focus of research in recent decades [36].

Drug repurposing, also known as drug repositioning, is a promising approach that identifies new therapeutic applications for already approved pharmaceuticals. This strategy has demonstrated success in treating various diseases, including Parkinson’s disease, depression, UC, fatty liver disease, breast cancer, irritable bowel syndrome, *H-pylori*, and colon cancer [37,38,39,40,41,42,43,44].

Statins, widely recognized as potent inhibitors of cholesterol biosynthesis, play a crucial role in the primary and secondary prevention of coronary heart disease due to their high safety profile and tolerability. Beyond their cardiovascular benefits, emerging evidence suggests that statins may contribute to UC management through their anti-inflammatory, immunomodulatory, and antioxidant properties [45]. While several animal studies have reported the positive effects of statins on IBD outcomes, the precise mechanism of action remains incompletely understood. Further research is needed to elucidate the pathways through which statins exert their potential therapeutic effects in IBD [21,24,25,46].

A randomized clinical study indicated that atorvastatin did not demonstrate significant benefits in managing acute UC exacerbations. Moreover, some patients experienced a paradoxical worsening of disease severity, highlighting the need for further investigation into its potential role and underlying mechanisms in UC treatment [47]. In this study, some patients reported increased disease activity following atorvastatin administration; however, these findings were not consistently supported by objective measures such as histology, endoscopic evaluation, or inflammatory biomarkers. Additionally, multiple studies suggest that high-dose atorvastatin (80 mg/day) is more effective in reducing inflammation compared to lower doses. Notably, patients receiving the higher dose exhibited a significant reduction in inflammatory markers, reinforcing the potential dose-dependent anti-inflammatory effects of atorvastatin [48,49]. Furthermore, Grip et al. reported that atorvastatin (80 mg daily) had a notable impact on both clinical symptoms and inflammatory markers in CD, suggesting its potential therapeutic role in managing inflammation in IBD [50]. Thus, the use of a low atorvastatin dose (20 mg/day) and the short study duration (two months) may have contributed to the lack of efficacy observed in the previous study. These factors might have been insufficient to elicit significant anti-inflammatory effects or mitigate UC exacerbation.

To the best of our knowledge, this is the first clinical pilot study to assess the impact of atorvastatin on DAI, serum zonulin, and fecal myeloperoxidase in UC patients. Previous research has explored and validated the potential efficacy of atorvastatin in UC management [24,51]. Moreover, retrospective cohort study has indicated that statin therapy in patients with IBD is associated with a reduction in corticosteroid usage, suggesting a potential role for statins in mitigating disease flares and minimizing reliance on immunosuppressive treatments [52]. The atorvastatin group showed a significant decrease in the DAI, bleeding, and diarrhea ratings when compared to the placebo group. This improvement is likely due to the combined anti-inflammatory effects of mesalamine and atorvastatin, which contribute to alleviating gastrointestinal symptoms. These findings align with previous research highlighting the potential therapeutic benefits of statins in IBD [26,53,54]. Our results support the notion that atorvastatin may aid patients with mild to moderate UC by reducing their symptoms. In experimental colitis models, atorvastatin administration significantly reduced colonic endoscopic scores and improved histological and immunohistochemical parameters compared to pre-treatment assessments. These results suggest a potential role for atorvastatin in modulating intestinal inflammation and promoting mucosal healing in UC [26]. El-Mahdy et al., reported that co-administration of atorvastatin plus mesalamine markedly attenuated DAI in oxazolone induced colitis [26]. In this study reported by El-Mahdy et al., they noticed that animal colitis treated with atorvastatin have higher levels of ant-inflammatory cytokine (IL-10), indicating the anti-inflammatory effect of statin. They also revealed that atorvastatin significantly elevated and upregulated tight junction proteins [26]. The preventive effects of atorvastatin in lowering DAI and decreasing inflammation in UC patients were all demonstrated by these findings.

The combination of mesalamine and atorvastatin resulted in a more pronounced suppression of myeloperoxidase activity compared to monotherapy. Myeloperoxidase, a lysosomal enzyme, is released into the neutrophil phagosome during degranulation, where it catalyzes the production of hypochlorous acid from hydrogen peroxide and halides, as well as tyrosyl radicals from tyrosine. These reactive species contribute to oxidative stress, tissue injury, and inflammation, highlighting the potential therapeutic benefit of dual therapy in mitigating intestinal inflammation in UC [55]. Myeloperoxidase levels are increased in various inflammatory conditions, including IBD, where it serves as a key biomarker of neutrophil activity and disease severity. Its elevated presence in the intestinal mucosa and feces reflects heightened inflammation and oxidative stress, making it a valuable indicator for monitoring disease progression and response to therapy [56]. Consequently, myeloperoxidase holds promise as a novel, non-invasive fecal or colonic biomarker for assessing IBD severity. Its presence in stool samples provides a practical and reliable means of monitoring disease activity, aiding in both diagnosis and treatment evaluation without the need for invasive procedures [57]. Our study’s findings are compatible with those of several other investigations on mucosal inflammation [58,59]. The present study demonstrated that the addition of atorvastatin to mesalamine therapy resulted in a significantly greater reduction in myeloperoxidase activity compared to mesalamine alone, aligning with findings from previous studies [26,60]. Malekinejad et al. suggested that atorvastatin reduces myeloperoxidase activity via a peroxisome proliferator-activated receptor gamma (PPAR)-γ-dependent pathway. This mechanism highlights the potential role of atorvastatin in modulating inflammatory responses in UC [60]. Others have linked atorvastatin’s anti-myeloperoxidase effects to its ability to modulate Toll-like receptor 4 (TLR-4) expression. This suggests that atorvastatin may exert its anti-inflammatory properties by influencing innate immune signaling pathways [61]. 

According to our findings, serum zonulin levels were significantly lower in the group receiving the combination of atorvastatin and mesalamine compared to both the mesalamine-only group and baseline values, highlighting the potential additive effect of atorvastatin on intestinal barrier function. These results are consistent with those of Caviglia et al., who found that IBD patients have higher levels of serum zonulin than healthy people. Serum zonulin is a crucial regulator of intercellular junction proteins that maintain intestinal permeability [62]. Additionally, compared to mesalamine alone, El-Mahdy et al. found that the combination of mesalamine plus the anti-inflammatory medication metformin considerably lowers serum zonulin [63]. Additionally, zonulin is associated with elevated levels of pro-inflammatory cytokines, which contribute to mucosal inflammation and exacerbate disease severity in IBD [64]. This breach in barrier integrity due to zonulin overexpression leads to an uncontrolled passage of dietary and microbial antigens across the intestinal epithelium, triggering immune activation and sustained inflammation [65]. Atorvastatin upregulated tight junction proteins and decreases intestinal permeability as illustrated by previous studies [66,67].

Since there were no significant differences in demographic or clinical variables between the groups at baseline, the therapeutic effects observed in this study can be attributed primarily to the administered medications. Notably, the placebo group demonstrated a significant reduction in serum zonulin and fecal myeloperoxidase levels compared to baseline. Additionally, DAI in this group was markedly lower than its initial value. These effects were likely due to mesalamine, a well-established treatment for mild to moderate UC, known for its anti-inflammatory properties [29,40,68]. These findings align with previous studies that have examined the impact of mesalamine on zonulin and myeloperoxidase levels in animal models of colitis. Research has consistently shown that mesalamine helps restore intestinal barrier integrity by reducing zonulin expression and mitigates neutrophil-driven inflammation by suppressing myeloperoxidase activity [23,26]. Mesalamine exerts its anti-inflammatory and apoptotic effects through a PPAR-gamma-dependent mechanism, which inhibits the production of pro-inflammatory cytokines. This pathway plays a crucial role in regulating immune responses and maintaining intestinal homeostasis, thereby contributing to the therapeutic benefits of mesalamine in UC management [69]. According to El-Haggar et al., mesalamine substantially lowered zonulin and other inflammatory mediators in patients with mild to moderate UC [28].

A significant correlation was observed between fecal myeloperoxidase and the DAI, as well as between fecal myeloperoxidase levels and serum zonulin. These findings suggest that a reduction in myeloperoxidase levels contributes to the attenuation of UC and a decrease in disease activity. Consequently, the decline in disease activity, fecal myeloperoxidase, and serum zonulin levels collectively results in a lower disease score. Several studies have similarly demonstrated that fecal myeloperoxidase is strongly associated with disease activity in patients with IBD, reinforcing its potential as a reliable biomarker for monitoring disease severity [56,70,71]. Previous research on myeloperoxidase in IBD identified a threshold of 7 μg/g as the optimal cutoff for detecting endoscopic disease activity. Collectively, these findings highlight the role of neutrophil-derived biomarkers, such as myeloperoxidase, in reflecting the inflammatory burden of the gut. This inflammatory burden is a key driver of the long-term progression of IBD, emphasizing the potential of myeloperoxidase as a valuable biomarker for disease monitoring and management [1]. Neutrophil activation is a critical factor in perpetuating the dysregulated innate immune responses seen in IBD. As a result, fecal biomarkers like myeloperoxidase may serve as reliable surrogate indicators of disease activity at a given moment. Additionally, these biomarkers could aid in stratifying overall disease prognosis, offering valuable insights into the severity and progression of IBD [72].

## 5. Conclusions

This clinical study directly compared the effects of mesalamine monotherapy with those of a combination of mesalamine and atorvastatin on DAI, fecal myeloperoxidase, and serum zonulin levels in patients with UC. The observed reductions in these biomarkers in the combination group highlight the potential additive anti-inflammatory and barrier-protective effects of atorvastatin when used alongside mesalamine. These findings support the role of atorvastatin as a promising adjunctive therapy in the management of UC, potentially enhancing the therapeutic outcomes of standard treatment.

Despite the study’s limitations, including a short follow-up period, a relatively small sample size, and the absence of varied atorvastatin doses, our findings highlight the need for further investigation. We encourage larger-scale randomized clinical trials with extended durations and larger participant groups to validate these results.

Comparing UC patients with healthy controls could provide additional insights; however, in Egypt, legal restrictions prevent the enrollment of healthy volunteers in research trials. Additionally, assessing creatine kinase enzyme levels and lipid profiles post-treatment would have been beneficial in evaluating the broader effects of atorvastatin.

A notable limitation of this study is the absence of colonoscopy and histological assessment, as disease severity was primarily assessed using DAI and other biomarkers. The reluctance of patients to undergo invasive procedures like colonoscopy underscores the ongoing need for improved, non-invasive diagnostic tools for UC.

## Figures and Tables

**Table 1:** Effect of study medications on disease activity index.

	Placebo Group (n = 24)			Atorvastatin Group (n = 23)			*p* Value
Character	Before Treatment	After Treatment	*p* Value	Before Treatment	After Treatment	*p* Value	After Treatment
Bleeding score	1 (1–1)	1 (0–1)	0.003 *	1 (1–2)	0 (0–1)	0.0007 *	0.03 **
Diarrhoea score	2 (2–3)	1 (0–1.75)	0.001 *	2 (1–3)	0 (0–75)	0.0009 *	0.04 **
Haemoglobin (mg/mL)	11.57 ± 0.936	13.99 ± 0.891	0.004 ^#^	12.17 ± 1.46	15.48 ± 1.28	0.0007 ^#^	0.003 ^##^
Albumin (g/dL)	4.040 ± 0.738	5.104 ± 0.560	0.03 ^#^	4.227 ± 0.750	5.101 ± 0.751	0.02 ^#^	0.986
ESR	22.38 ± 4.13	12.21 ± 2.91	<0.0001 ^#^	23.04 ± 3.77	9.870 ± 3.09	<0.0001 ^#^	0.012 ^##^
DAI	201.2 (187.6–208)	127.8 (82.5–141.9)	<0.0001 *	192.4 (180.4–213.7)	73.45 (57.2–89.67)	<0.0001 *	0.001 **

Data are presented as mean ±SD, median, and interquartile range, Placebo group, UC patients treated with mesalamine and placebo, atorvastatin group, UC patients treated with mesalamine plus atorvastatin, ESR, erythrocyte sedimentation rate, DAI, disease activity index. (^#^) Level of significance within the same group by paired *t*-test. (^##^) Level of significance between groups by an unpaired *t*-test. (*) Level of significance within the same group by Wilcoxon test. (**) Level of significance between groups by Man–Whitney test. Statistical significance was set at (*p* < 0.05).

**Table 2:** Effect of study medications clinical outcomes.

Character	Placebo Group (n = 24)			Atorvastatin Group (n = 23)			*p* Value
	Before Treatment	After Treatment	*p* Value	Before Treatment	After Treatment	*p* Value	After Treatment
Zonulin (ng/mL)	57.65 (52.25–64.05)	42.85 (31–57.75)	0.01 *	59.40 (37.5–63.5)	27.9 (12.5–53.4)	0.0007 *	0.04 **
Myeloperoxidase (ng/mL)	23.35 (20.9–24.3)	16.25 (14.34–17.33)	0.0002 *	23.65 (11.2–24.8)	13.36 (10.8–16.38)	0.0005 *	0.03 **

Data are presented as median and interquartile range, placebo group, UC patients treated with mesalamine and placebo, atorvastatin group, and UC patients treated with mesalamine plus atorvastatin. (*) level of significance within the same group using the Wilcoxon test. (**) Level of significance between groups using the Mann–Whitney test. Statistical significance was set at (*p* < 0.05).

## Data Availability

All data are available from the corresponding author upon reasonable request.

## Supplementary Materials

The following supporting information can be downloaded at: https://www.mdpi.com/article/10.3390/jcm14093077/s1, Table S1: Clinical, demographic and laboratory data of the patients.
