# Supplementary material for: Repurposing Atorvastatin, HMGCO-A Reductase Inhibitor, in Patients with Ulcerative Colitis: A Randomized Controlled Study
Source: J Clin Med. 2025 Apr 29;14(9):3077. doi: 10.3390/jcm14093077 (PMC12072543; doi:10.3390/jcm14093077)
Supplement: Supplementary file 1 [file jcm-14-03077-s001.zip › jcm-3571430-supplementary.pdf]

Table S1: Clinical, demographic and laboratory data of the patients.

Parameter	Placebo group (n=29)	Atorvastatin group (n=27)	P value
Age (years)	44.14 ± 14.31	45.22 ± 11.77	0.759
Sex (M/F)	16 /13	13 /14	0.599
BMI (kg/m^2)	23.21 ± 1.571	23.65 ± 1.142	0.242
Serum ALT (IU/L)	44.59 ± 11.58	46.67 ± 9.872	0.474
Serum AST (IU/L)	42.86 ± 10.75	47.15 ± 13.61	0.194
Hgb (g/dl)	11.73 ± 1.011	12.16 ± 1.450	0.204
Albumin (g/dl)	4.004 ± 0.679	4.140 ± 0.747	0.479
Disease durations (year)	1.6 (0.9-2.6)	1.8 (0.9-2.7)	0.445
SrCr (mg/dl)	0.877 ± 0.193	0.816 ± 0.144	0.186
TC (mg/dl)	162.4 ± 14.48	165.5 ± 13.46	0.411
TG (mg/dl)	133.1 ± 11.37	130.5 ± 11.61	0.391
LDL (mg/dl)	92.23 ± 15.59	94.42 ± 14.59	0.590
HDL (mg/dl)	43.52 ± 6.9	44.96 ± 8.022	0.471
Smoking (n)	6	4	0.566

Data are presented as mean ±SD, numbers and interquartile range; placebo group, UC patients treated with mesalamine and placebo; atorvastatin group, UC patients treated with mesalamine plus Atorvastatin, M: Male, F: Female, ALT, alanine aminotransferase; AST, aspartate aminotransferase; Hgb, hemoglobin; Sr, Cr, serum creatinine; (TC), total cholesterol; (TG), triglycerides; (HDL), high-density lipoprotein; (LDL), low-density lipoprotein. Statistical significance was set at ($p < 0.05$).
